# The impact of subclinical hypothyroidism on long-term outcomes in older patients undergoing percutaneous coronary intervention

**DOI:** 10.1186/s12902-021-00702-z

**Published:** 2021-03-05

**Authors:** Yong-Sheng Liu, Mei Wei, Le Wang, Gang Liu, Guo-Ping Ma, Katsushige Ono, Ze-Long Cao, Man Yang, Ming-Qi Zheng

**Affiliations:** 1 Heart Center, The First Hospital of Hebei Medical University, No.89 Donggang Road, Shijiazhuang, 050031 Hebei China; 2 Department of Cardiology, Tianjin Chest Hospital, Tianjin, 300222 China; 3 Department of Pathophysiology, Oita University School of Medicine, Yufu, Oita 879-5593 Japan

**Keywords:** Subclinical hypothyroidism, Mortality, Older, Percutaneous coronary intervention

## Abstract

**Background:**

Subclinical hypothyroidism (SCH) is reportedly associated with an increased risk of adverse events in patients undergoing percutaneous coronary intervention (PCI). The prognostic significance of SCH in the elderly was poorly defined. The purpose of this study was to evaluate the association between SCH and long-term outcomes in older patients undergoing PCI.

**Methods:**

Three thousand one hundred sixty-eight patients aged 65 years or older who underwent PCI from January 2012 to October 2014 were included. Patients were divided into SCH group (*n* = 320) and euthyroidism (ET) group (*n* = 2848) based on thyroid function test. Cox proportional hazard regression analyses were used to estimate the relative risks (RRs) of all-cause death and cardiac death for patients with SCH during a 4-year follow-up period.

**Results:**

There were 227 deaths during the follow-up period including 124 deaths caused by cardiac events. There was no significant difference in mortality rate between the SCH group and the ET group (*p* > 0.05). After adjustment for covariates, compared with patients with ET, the RRs of death from all-cause and cardiac in patients with SCH were 1.261 (95%CI: 0.802–1.982, *p* = 0.315) and 1.231 (95%CI: 0.650–2.334, *p* = 0.524), respectively. When SCH was stratified by age, gender, and degree of thyroid-stimulating hormone elevation, no significant associations were also found in any stratum.

**Conclusion:**

Our investigation revealed that SCH was negatively associated with the outcome of PCI in older patients.

## Background

Nowadays elderly patients receiving the percutaneous coronary intervention (PCI) represent more than one in five patients treated with PCI in real-world practice [1]. Mortality remained markedly higher in elderly patients than those in younger counterparts after PCI [2]. The prediction of death after PCI is a challenging task for clinicians. To further improve the prognosis of elderly patients after PCI, significant attention has been paid to identify modifiable risk factors of mortality.

Increased risk of cardiovascular diseases including atherosclerosis are found among subclinical hypothyroidism (SCH) patients, whose thyroid hormone levels remain normal and only thyroid-stimulating hormone (TSH) levels are increased. Some studies suggest that SCH is associated with hypercholesterolemia and atherosclerosis, and it should be an independent risk factor for atherosclerosis and myocardial infarction. Besides these, Thyroid hormones have other different effects on the cardiovascular system. Subclinical hypothyroidism (SCH) has been associated with unfavorable effects, such as worsening blood pressure [3], atherogenic dyslipidemia [4], impaired cardiac contractile and diastolic function [5], increased systemic vascular resistance [6], endothelial dysfunction [6], accelerated atherosclerosis [7], enhanced inflammation level [8], hypercoagulability, insulin resistance and oxidative stress [7], all of which may increase the risk of death. Up to 10% of the elderly have SCH, which is usually asymptotic [9]. The high prevalence of SCH in the elderly has led to a significant body of research concerning the possibility that SCH may herald mortality. However, current evidence on the association between SCH and mortality in the elderly are conflicting [10–14].

To date, reliable information on the association between SCH and mortality in older patients with coronary artery disease is limited. Whether SCH contributes to mortality in elderly patients undergoing PCI remains unclear. Therefore, to further examine the association between SCH and risk of all-cause as well as cardiac mortality in elderly patients, we evaluate the effect of SCH on mortality rate in patients aged 65 years or older who underwent PCI in a single high-volume center.

## Methods

### Study population and follow-up

**Fig. 1:**
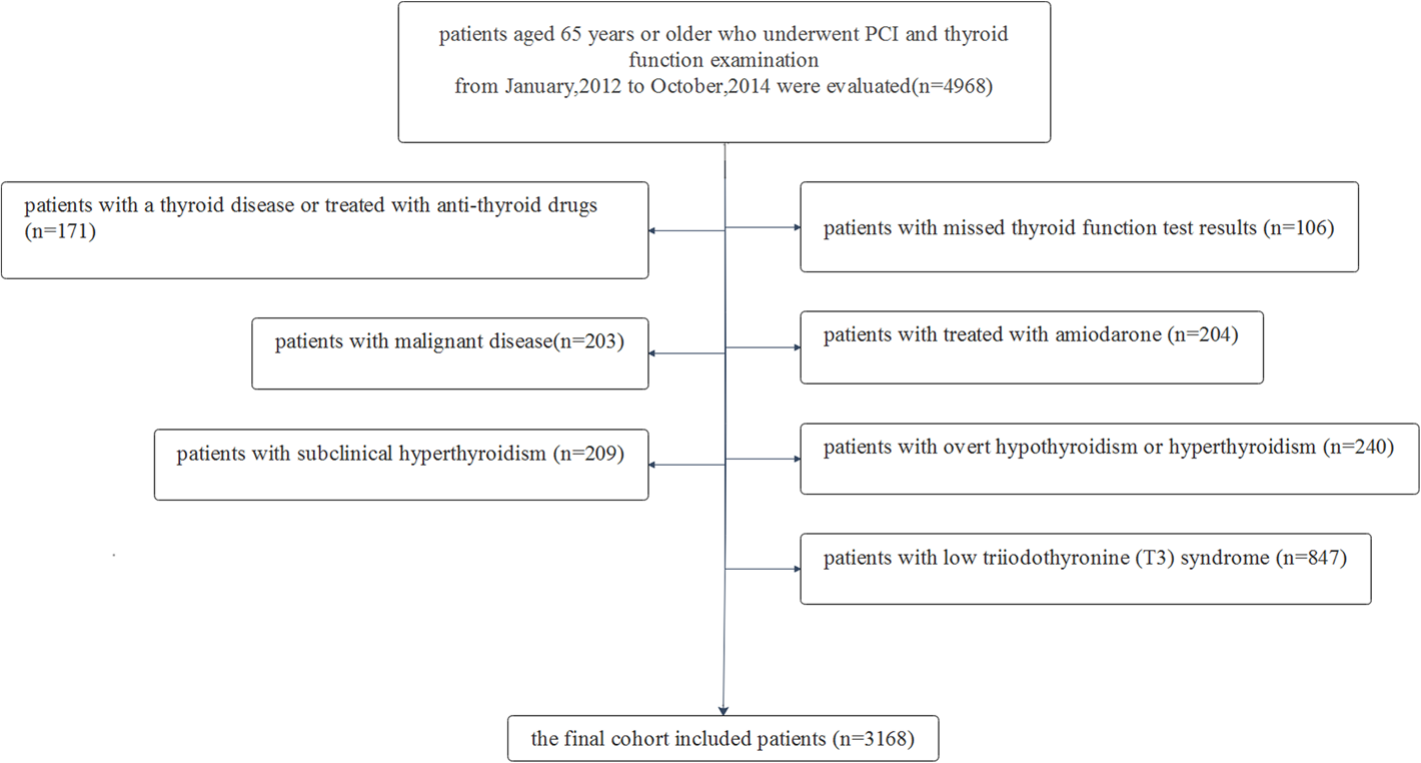
Patients included in the study, excluded from the study and follow-up criteria

This was a retrospective cohort study from January 2012 to October, 2014. We evaluated 4968 consecutive patients aged 65 years or older who underwent PCI and thyroid function examination at First Affiliated Hospital of Hebei Medical University. The following patients were excluded from the study: 106 patients with missed thyroid function test results; 171 patients with a thyroid disease or treated with anti-thyroid drugs; 204 patients treated with amiodarone; 23 patients with malignant disease; 240 patients with overt hypothyroidism or hyperthyroidism; 209 patients with subclinical hyperthyroidism; 847 patients with low triiodothyronine syndrome. Thus, the final cohort included 3168 patients. All the patients’ information was obtained by independent reviewers who were blind to the purpose of the study. All clinical, laboratory, medication and PCI data were collected. Clinical follow-up was performed by either telephone contact or office visit. All the patients were prospectively followed up for 4 years and the follow-up rate was 90.1%. The end point of this study was all-cause mortality and cardiovascular disease (CVD)-related death. Causes of death were determined by medical records, death certificates and autopsy reports. All the patients in the retrospective study gave written informed consent (Fig. 1).

### Thyroid function testing

Thyroid function test was performed by after hospital admission and before PCI. Serum TSH, total triiodothyronine (TT3), total thyroxine (TT4), free triiodothyronine (FT3) and free thyroxine (FT4) levels were measured by chemiluminescence immunoassay. The reference intervals for thyroid function test were TT4, 78.43–157.40 nmol/L; TT3, 1.34–2.73 nmol/L; TSH, 0.34–5.60 mIU/L; FT3, 3.80–6.00 pmol/L; FT4, 7.90–14.40 pmol/L. Euthyroidism (ET) was defined as all circulating level of TSH, FT3, FT4, TT3 and TT4 in the normal range. Subclinical hypothyroidism (SCH) was defined as TSH > 5.60 mIU/L, with FT3, FT4, TT3 and TT4 in the normal range, without symptoms or signs of hypothyroidism.

### Statistical analysis

Continuous variables were expressed as mean ± standard deviation when normally distributed and as medians with inter quartile ranges for results not normally distributed. Categorical variables were presented as frequencies. Baseline clinical, laboratory, medication and PCI data between groups were compared using unpaired Student’s t-test or Mann-Whitney U test for continuous variables and chi-square test or Fisher exact test for categorical variables. Kaplan-Meier survival curves associated with subclinical hypothyroidism and euthyroidism were compared with log-rank test. Multivariable Cox proportional hazards regression analysis was performed to estimate hazard ratios (HR) for all-cause death and cardiac death. We calculated multivariate HRs by adjusting for age, gender, body mass index, hypertension, diabetes mellitus, hyperlipidemia, smoking, family history of coronary artery disease, history of myocardial infarction, history of percutaneous coronary intervention, history of the coronary artery bypass graft, history of stroke, history of heart failure, history of renal failure, acute myocardial infarction, left ventricle ejection fraction, hemoglobin, fasting glucose, creatinine, total cholesterol, triglyceride, low density lipoprotein cholesterol, high density lipoprotein cholesterol, high-sensitivity C-reactive protein, aspirin, clopidogrel, ß-Blocker, angiotensin II coenzyme inhibitor, angiotensin II receptor blocker, statins, multi-vessel disease, left main, left anterior descending, left circumflex artery, right coronary artery. A 2-sided analysis with a *P* value < 0.05 was considered significant. All analyses were performed using the SPSS software program, version25 (IBM Corp. Released 2017. IBM SPSS Statistics for Windows, Version 25.0. Armonk, NY: IBM Corp.)

## Results

### Patients characteristics

The cohort consisted of 3168 older patients who underwent PCI. Of the 3168 patients, 10.1% (*n* = 320) had SCH, 89.9% (*n* = 2848) had ET. The baseline clinical, biological and medication characteristics are summarized in Table 1. Compared with the patients with ET, SCH was more common in female patients and patients with hyperlipidemia, and it was associated with higher body mass index, lower hemoglobin level, higher serum total cholesterol, triglyceride, low density lipoprotein levels and lower high-density lipoprotein level. Frequency of antianginal drugs, antiplatelet agents and lipid lowering medicine was similar between the two groups. The angiographic and PCI data are shown in Table 2. There were no significant differences in the extent of diseased vessels, targeted vessel distribution, and number of stents between the two groups.

**Table 1:** Baseline clinical, biological, and medication characteristics

	SCH (***n*** = 320)	ET (***n*** = 2848)	***P*** Value
Clinical characteristics			
Age, years	70.4 ± 4.0	70.9 ± 4.2	0.038
Female, n (%)	186 (58.1)	1152 (40.4)	0.000
BMI, kg/m^2^,	26.4 ± 3.2	25.5 ± 3.0	0.000
Hypertension, n (%)	248 (77.5)	2128 (74.7)	0.276
Diabetes mellitus, n (%)	100 (31.3)	932 (32.7)	0.594
Hyperlipidemia, n (%)	174 (54.4)	1306 (45.9)	0.004
Current smoking, n (%)	58 (18.1)	572 (20.1)	0.405
Family history of CAD, n (%)	16 (5.0)	174 (6.1)	0.428
History of MI, n (%)	28 (8.8)	352 (12.4)	0.060
History of PCI, n (%)	44 (13.8)	496 (17.4)	0.098
History of CABG, n (%)	4 (1.3)	74 (2.6)	0.140
History of Stroke, n (%)	36 (11.3)	358 (12.6)	0.497
History of HF, n (%)	26 (8.1)	177 (6.2)	0.186
History of RF, n (%)	12 (3.8)	136 (4.8)	0.410
SAP, n (%)	40 (12.5)	414 (14.5)	0.324
UAP, n (%)	216 (67.5)	1928 (67.7)	0.943
AMI, n (%)	64 (20.0)	506 (17.8)	0.324
LVEF, (%)	63.6 ± 7.8	63.0 ± 9.1	0.265
Laboratory characteristics			
TSH	7.19 (6.20–8.28)	1.64 (1.10–2.46)	0.000
FT3	4.80 (4.20–5.40)	4.90 (4.30–5.40)	0.301
FT4	11.00 (9.33–12.80)	11.00 (9.40–12.70)	0.879
Hemoglobin, g/dl	129 (123, 142)	137 (127, 147)	0.000
Fasting glucose, mmol/L	5.57 (5.03, 6.49)	5.62 (5.09, 6.51)	0.478
Creatinine, μmol/L	74.5 (62.0, 89.0)	76.0 (65.0, 89.0)	0.206
TC, mmol/L	4.50 (4.04, 5.15)	4.30 (3.64, 5.01)	0.000
TG, mmol/L	1.50 (1.56, 1.97)	1.39 (1.03, 1.88)	0.002
LDL-C, mmol/L	2.61 (2.25, 3.11)	2.58 (2.01, 3.11)	0.018
HDL-C, mmol/L	1.14 (1.01, 1.34)	1.09 (0.94, 1.26)	0.000
HsCRP, mg/L	2.00 (0.71, 4.83)	1.75 (0.69, 4.60)	0.221
Medications at discharge			
Aspirin, n (%)	318 (99.4)	2842 (99.8)	0.416
Clopidogrel, n (%)	318 (99.4)	2843 (99.8)	0.319
ß-Blocker, n (%)	256 (80.0)	2200 (77.2)	0.263
ACEI/ARB, n (%)	174 (54.4)	1562 (54.8)	0.873
Calcium channel antagonist, n (%)	102 (31.9)	796 (27.9)	0.140
Nitrates, n (%)	198 (61.9)	1866 (65.5)	0.195
Statins, n (%)	308 (96.3)	2732 (95.9)	0.781

Data are expressed as mean ± SD, medians with inter quartile ranges or percentage. *BMI* body mass index, *CAD* coronary artery disease, *MI* myocardial infarction, *PCI* percutaneous coronary intervention, *CABG* coronary artery bypass graft, *HF* hear failure, *RF* renal failure, *SAP* stable angina pectoris, *UAP* unstable angina pectoris, *AMI* acute myocardial infarction, *LVEF* left ventricle ejection fraction, *TC* total cholesterol, *TG* triglyceride, *LDL-C* low density lipoprotein cholesterol, *HDL-C* high density lipoprotein cholesterol, *HsCRP* high-sensitivity C-reactive protein, *ACE-I* angiotensin II coenzyme inhibitor, *ARB* angiotensin II receptor blocker

**Table 2:** Baseline angiographic and PCI characteristics

	SCH (***n*** = 320)	ET (***n*** = 2848)	***P*** Value
Diseased vessels			
1-vessel, n (%)	82 (25.6)	870 (30.5)	0.069
2-vessel, n (%)	100 (31.3)	836 (29.4)	0.481
3-vessel, n (%)	138 (43.1)	1142 (40.1)	0.348
Multi-vessel disease, n (%)	238 (74.4)	1978 (69.5)	0.069
Target vessel			
LM, n (%)	8 (2.5)	126 (4.4)	0.105
LAD, n (%)	174 (54.4)	1700 (59.7)	0.067
LCX, n (%)	92 (28.8)	846 (29.7)	0.723
RCA, n (%)	137 (42.8)	1128 (39.6)	0.267
Number of stents	1.8 ± 1.0	1.8 ± 1.0	0.898
Drug-eluting stent, n (%)	320 (100)	2848 (100)	1.000

Data are expressed as percentage. *LM* left main, *LAD* left anterior descending, *LCX* left circumflex artery, *RCA* right coronary artery

### Association between SCH and mortality

**Fig. 2:**
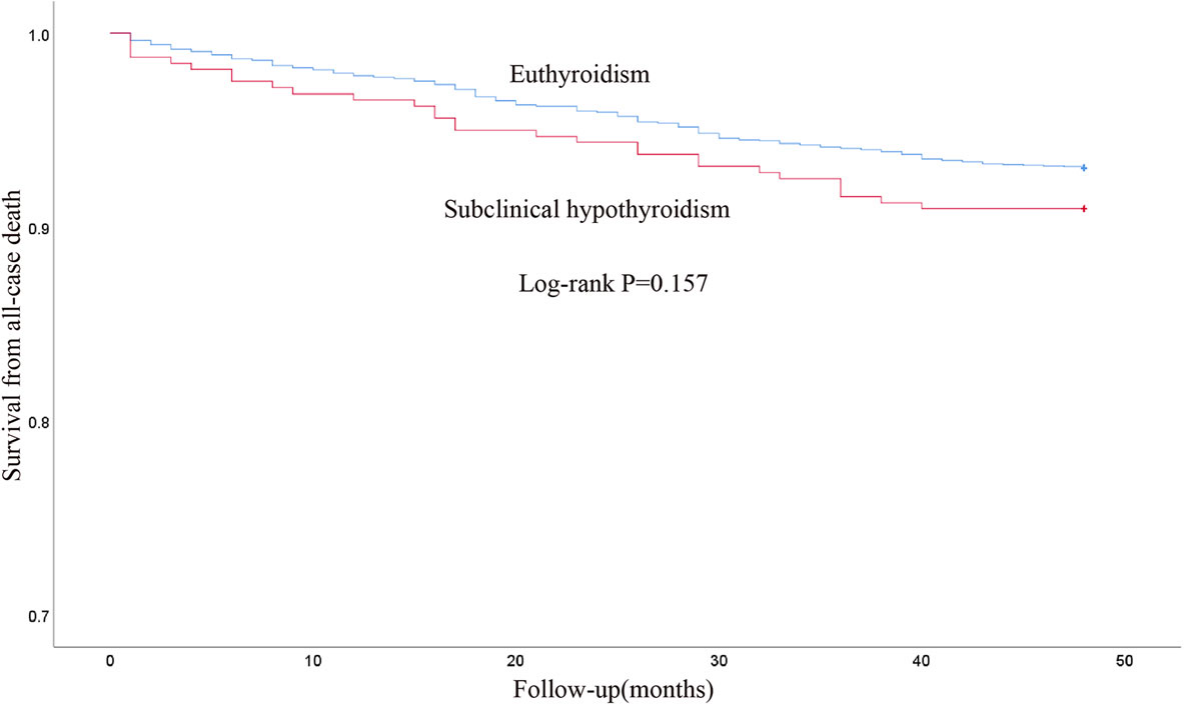
Kaplan-Meier survival curves for all-cause death for patients with SCH and ET

**Fig. 3:**
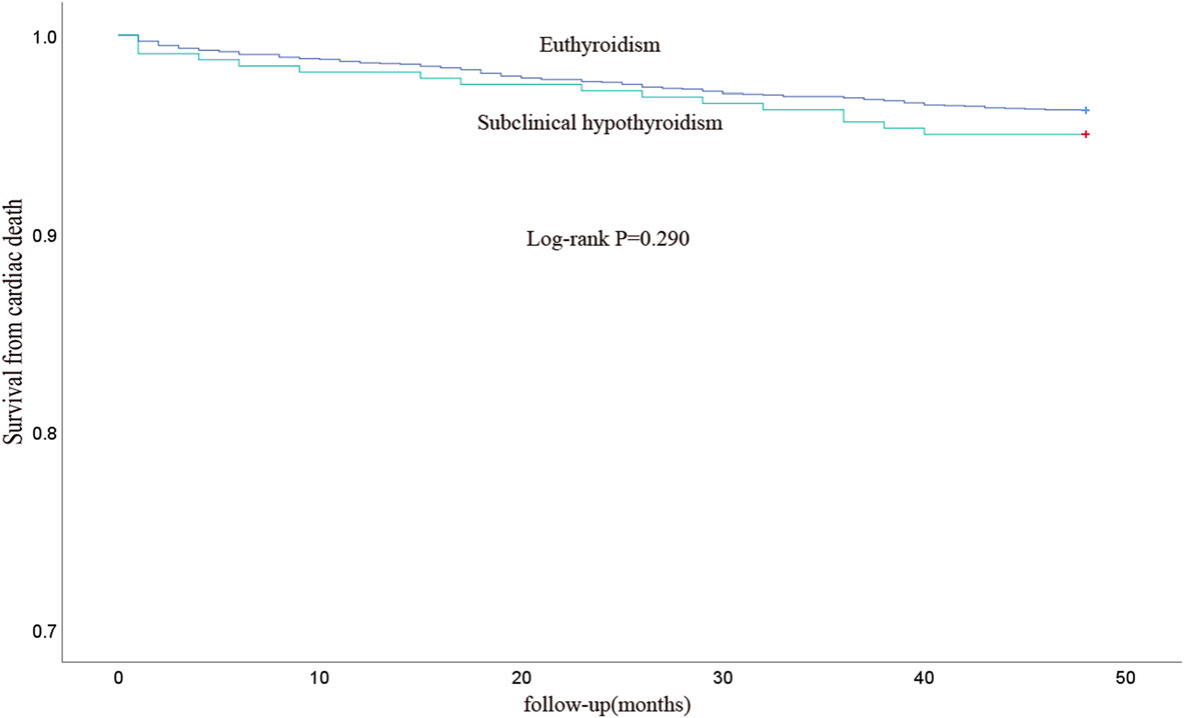
Kaplan-Meier survival curves for cardiac death for patients with SCH and ET

Among the 3168 patients, 227 patients died during 4-year follow-up. Of these, 29 were in SCH group and 198 in ET group. Among the 227 died patients, 124 patients died of cardiac cause. Cardiac death was 16 in the SCH group and 108 in the ET group. The 4-year cumulative all-cause mortality rates in patients with SCH and ET were 9.1 and 7.0% respectively. The 4-year cumulative cardiac mortality rates in patients with SCH and ET were 5.0 and 3.8% respectively. The Kaplan-Meier analysis showed that there was no significant difference in the all-cause mortality and cardiac mortality between patients with SCH and ET (Fig. 2 and Fig. 3).

### Relative risks for all-cause and cardiac death in patients with SCH vs. ET

Table 3 and Table 4 summarized the relative risks for all-cause and cardiac death in patients with SCH vs. ET. Compared with ET, SCH was not associated with higher risk of all-cause and cardiac death. Adjusted for age, gender, body mass index, hypertension, diabetes mellitus, hyperlipidemia, smoking, et al., compared with ET, SCH was not associated with a higher risk of all-cause and cardiac death in subgroup of different age, gender and TSH level.

**Table 3:** Relative risks for all-cause death in patients with SCH vs. ET

	Death(n)	Number at risk(n)	Unadjusted RR	95%CI	***P*** Value	Adjusted RR	95%CI	***P*** Value
ET	198	2848	1.000 (reference)			1.000 (reference)		
SCH	29	320	1.323	0.896–1.954	0.159	1.261	0.802–1.982	0.315
Age<75 years								
ET	159	2276	1.000			1.000		
SCH	25	268	1.356	0.889–2.067	0.157	1.261	0.767–2.074	0.360
Age ≥ 75 years								
ET	39	572	1.000			1.000		
SCH	4	52	1.138	0.407–3.185	0.805	0.295	0.029–2.963	0.300
Male								
ET	124	1696	1.000			1.000		
SCH	12	132	1.248	0.690–2.256	0.464	1.037	0.523–2.058	0.917
Female								
ET	74	1152	1.000			1.000		
SCH	17	186	1.444	0.852–2.446	0.172	1.016	0.500–2.065	0.965
TSH								
0.34–5.60	198	2848	1.000			1.000		
5.61–9.99	24	278	1.255	0.822–1.918	0.293	1.275	0.767–2.121	0.349
≥10	5	42	1.749	0.720–4.248	0.217	1.062	0.322–3.497	0.921

Adjusted RR: adjusted for age, gender, body mass index, hypertension, diabetes mellitus, hyperlipidemia, smoking, family history of coronary artery disease, History of myocardial infarction, history of percutaneous coronary intervention, history of the coronary artery bypass graft, history of stroke, history of heart failure, History of renal failure, acute myocardial infarction, left ventricle ejection fraction, hemoglobin, fasting glucose, creatinine, total cholesterol, triglyceride, low density lipoprotein cholesterol, high density lipoprotein cholesterol, high-sensitivity C-reactive protein, aspirin, clopidogrel, ß-Blocker, angiotensin II coenzyme inhibitor, angiotensin II receptor blocker, statins, multi-vessel disease, left main, left anterior descending, left circumflex artery, right coronary artery

**Table 4:** Relative risks for cardiac death in patients with SCH vs. ET

	Death(n)	Number at risk(n)	Unadjusted RR	95%CI	***P*** Value	Adjusted RR	95%CI	***P*** Value
ET	108	2848	1.000 (reference)			1.000 (reference)		
SCH	16	320	1.326	0.785–2.242	0.292	1.231	0.650–2.334	0.524
Age<75 years								
ET	83	2276	1.000			1.000		
SCH	14	268	1.445	0.820–2.545	0.203	1.304	0.639–2.661	0.467
Age ≥ 75 years								
ET	25	572	1.000			1.000		
SCH	2	52	0.876	0.208–3.700	0.858	0.762	0.324–1.678	0.938
Male								
ET	73	1696	1.000			1.000		
SCH	7	132	1.225	0.564–2.661	0.607	0.917	0.351–2.397	0.860
Female								
ET	35	1152	1.000			1.000		
SCH	9	186	1.602	0.770–3.333	0.207	1.0000	0.348–2.870	1.000
TSH								
0.34–5.60	108	2848	1.000			1.000		
5.61–9.99	13	278	1.238	0.696–2.201	0.467	1.054	0.508–2.187	0.887
≥10	3	42	1.934	0.614–6.092	0.260	1.321	0.274–6.358	0.729

Adjusted RR: adjusted for age, gender, body mass index, hypertension, diabetes mellitus, hyperlipidemia, smoking, family history of coronary artery disease, History of myocardial infarction, history of percutaneous coronary intervention, history of coronary artery bypass graft, history of stroke, history of heart failure, History of renal failure, acute myocardial infarction, left ventricle ejection fraction, hemoglobin, fasting glucose, creatinine, total cholesterol, triglyceride, low density lipoprotein cholesterol, high density lipoprotein cholesterol, high-sensitivity C-reactive protein, aspirin, clopidogrel, ß-Blocker, angiotensin II coenzyme inhibitor, angiotensin II receptor blocker, statins, multi-vessel disease, left main, left anterior descending, left circumflex artery, right coronary artery

## Discussions

In this large cohort of older patients aged 65 years or older undergoing PCI, SCH was not associated with increased risks of all-cause and cardiac mortality. The prognostic significance of SCH applied equally to the subgroup of different gender, age and thyroid function level. These results suggested that SCH may not confer the detrimental effect on older patients undergoing PCI.

The prevalence of SCH increases with age. In the present study, the prevalence of SCH was 10.1%, which was similar to previous studies [9]. SCH is associated with cardiovascular risk factors and metabolic syndrome [15]. A higher level of TSH has been related to increased BMI and hyperlipidemia [15]. Consistently, in the present study, we observed that patients with SCH have significant increases in BMI, serum level of TC, TG and LDL-C, which may explain why the patients in the SCH group received PCI at a younger age, comparing the ET group. As shown previously, SCH was more prevalent in female. In this cohort, women accounted for 58.1% in the SCH group. Also, the level of hemoglobin of patients with SCH was lower than that of patients with ET, which might be due to the higher female-to-male ratio in patients with SCH.

There are only a few studies on the relationship between SCH and mortality in the elderly. The prognostic significance of SCH in the elderly is controversial. Several studies reported no association of SCH with death from cardiovascular or all-cause mortality in the elderly [12, 13]. The Leiden 85+ study revealed that SCH was associated with decreased all-cause mortality and cardiovascular mortality [10]. In contrast, a recent retrospective study by Grossman et al. demonstrated that SCH is associated with increased mortality in the elderly [11]. A recent review reported that SCH was not associated with increased risk of cardiovascular mortality or total mortality in the elderly [16]. However, owing to the relatively few studies, the result should be interpreted carefully and confirmed by further studies. To date, most of the findings in the elderly were derived from the general population. Although SCH has been associated with an increased risk of mortality in patients with established cardiovascular disease [17, 18], it is ambiguously defined whether SCH increases the risk of mortality in older patients with established cardiovascular disease.

To date, only a few studies evaluated the special association of SCH with mortality in patients with a high risk of ischemic heart disease. A recent study by Zhang reported an association between SCH defined based on serum TSH level and major adverse cardiovascular and cerebral events in patients treated with PCI [19]. The results showed that cardiac death was significantly higher in patients with SCH compared with patients with ET. This finding was obtained in a population with a mean age of 64.6 years. Another recent study found that SCH defined based on serum TSH and FT4 levels was associated with cardiovascular events and cardiac death in patients following PCI [20]. In this study, unlike previous studies without restriction on age, we have evaluated a large cohort of patients aged 65 years or older treated with PCI and followed their mortality risk up to 4 years after PCI. In the present study, we defined SCH based on TSH, TT4, TT3, FT3, and FT4 levels to eliminate possible misclassification of SCH. We observed that there was no significant difference in mortality in elderly patients with SCH compared with patients with ET. In comparison with ET, SCH was not associated with a higher risk of all-cause and cardiac death. After adjustment for baseline variables, the prognostic significance of SCH applied equally to subgroups of different gender, different age and different degree of thyroid-stimulating hormone level. Our results suggest that SCH may not an independent risk factor for mortality in older patients undergoing PCI. The increase of serum TSH observed in the elderly may represent a physiological process reflecting a certain degree down-regulation hypothalamus-pituitary-thyroid-peripheral (HPTP) axis. Therefore, SCH, especially mild elevation of TSH, should not be regarded as a pathological condition in older patients undergoing PCI. Moreover, we focused end point on death to fully assess the impact of SCH on mortality in patients underwent PCI.

The association between SCH and mortality has been well established in young individuals [21]. SCH is associated with many well-known cardiovascular risk factors. However, we could not demonstrate the negative effect of SCH on mortality in older patients undergoing PCI. The presence of multiple morbidities in the elderly may contribute to the results. There are also several potential explanations for this neutral finding. First, the elderly patients and patients with SCH have common pathophysiological conditions. Additional assessment of serum TSH level adds little to the prediction ability of common risk scoring models, attesting to the fact that the mortality associated with SCH may be caused by traditional cardiovascular risk factors [22]. Thus these cardiovascular risk factors for CHD among those elderly patients undergoing PCI may overshadow the negative effect of SCH on cardiovascular system. Second, an interaction between age and SCH cannot be refuted. A recent cohort study on 80,490 persons found that the association between SCH between all-cause or vascular mortality was stronger in men below 60 years compared to older males [23]. Another meta-analysis showed that the risk of the cardiovascular and all-cause mortality is higher in those mean age < 65 years than for those of average age ≥ 65 years [16]. All these results suggest that there may be age-related mortality difference associated with SCH, with stronger association in younger patients that attenuated advancing age. In addition, the current evidence to make a recommendation for levothyroxine therapy is not strong. Levothyroxine treatment in patients with SCH and established heart disease was not associated with a significant benefit for the risk of all-cause mortality [24]. A recent prospective study found no beneficial effect of treatment with levothyroxine in older adults [25]. Moreover, another study reported that treatment with levothyroxine was associated with excess mortality in individuals 65 years or older with SCH [26]. Taken together, it is suggested that SCH may not have negative impact on mortality in the elderly.

### Limitations

The present study has several limitations. First, the present study was a single-center retrospective study, and replication is needed to assess the validity of the findings, although the number of the patients analyzed in the present study are large. Second, thyroid function test was only performed at baseline, and the influences of medication treatments on thyroid function and the natural history of SCH were not investigated. However, compared with previous studies, we simultaneously measured serum FT3, FT3, T3, T4 and TSH levels. We defined SCH strictly. Therefore, the present study truly reflected the effect of SCH on mortality in older patients after PCI. Moreover, previous study did not demonstrate increased risk of cardiovascular death in older adult with persistent SCH [12]. Third, previous studies revealed that SCH in patients with an acute cardiac disease have been associated with increased risk of death. Due to the limited number of patients, we did not make subgroup analysis according to the clinical presentation. Thyroxine in acute myocardial infarction study will clarify the association of thyroid function at the time of AMI with cardiovascular outcomes [27]. Finally, our study revealed no increased risk of mortality in patients with TSH ≥ 10 mIU/L. The small sample size with TSH ≥ 10 mIU/L may weaken the reliability of the result. Therefore, the result needs to be further confirmed in large prospective studies.

## Conclusions

SCH on admission was not associated with higher risk of all-cause death and cardiac death in older patients undergoing PCI. Our results suggest that SCH does not represent a risk factor for morality in patients aged 65 or older undergoing PCI.

## Data Availability

The raw/processed data required to reproduce these findings cannot be shared for now, because these data are part of ongoing follow-up studies. The datasets used and analysed during the current study are available from the corresponding author on reasonable request.

## Abbreviations

SCH: Subclinical hypothyroidism
PCI: Percutaneous coronary intervention
ET: Euthyroidism
RRs: Relative risks
TSH: Thyroid-stimulating hormone
CVD: Cardiovascular disease
TT3: Total triiodothyronine
TT4: Total thyroxine
FT3: Free triiodothyronine
FT4: Free thyroxine
ET: Euthyroidism
BMI: Body mass index
CAD: Coronary artery disease
MI: Myocardial infarction
CABG: Coronary artery bypass graft
HF: Hear failure
RF: Renal failure
SAP: Stable angina pectoris
UAP: Unstable angina pectoris
AMI: Acute myocardial infarction
LVEF: Left ventricle ejection fraction
TC: Total cholesterol
TG: Triglyceride
LDL-C: Low density lipoprotein cholesterol
HDL-C: High density lipoprotein cholesterol
HsCRP: High-sensitivity C-reactive protein
ACE-I: Angiotensin II coenzyme inhibitor
ARB: Angiotensin II receptor blocker
LM: Left main
LAD: Left anterior descending
LCX: Left circumflex artery
RCA: Right coronary artery

## References

[1] Shanmugam VB, Harper R, Meredith I, Malaiapan Y, Psaltis PJ (2015). An overview of PCI in the very elderly. J Geriatr Cardiol.

[2] Vandermolen S, Abbott J, De Silva K (2015). What's age got to do with it? A review of contemporary revascularization in the elderly. Curr Cardiol Rev.

[3] Ye Y, Xie H, Zeng Y, Zhao X, Tian Z, Zhang S (2014). Association between subclinical hypothyroidism and blood pressure-a meta-analysis of observational studies. Endocr Pract.

[4] Pearce EN (2012). Update in lipid alterations in subclinical hypothyroidism. J Clin Endocrinol Metab.

[5] Masaki M, Komamura K, Goda A, Hirotani S, Otsuka M, Nakabo A (2014). Elevated arterial stiffness and diastolic dysfunction in subclinical hypothyroidism. J Circ J.

[6] Razvi S, Weaver JU, Pearce SH (2010). Subclinical thyroid disorders: significance and clinical impact. J Clin Pathol.

[7] Biondi B, Cooper DS (2008). The clinical significance of subclinical thyroid dysfunction. Endocr Rev.

[8] Marfella R, Ferraraccio F, Rizzo MR, Portoghese M, Barbieri M, Basilio C (2011). Innate immune activity in plaque of patients with untreated and L-thyroxine-treated subclinical hypothyroidism. J Clin Endocrinol Metab.

[9] Floriani C, Gencer B, Collet TH, Rodondi N (2017). Subclinical thyroid dysfunction and cardiovascular diseases: 2016 update. Eur Heart J.

[10] Gussekloo J, van Exel E, de Craen AJ, Meinders AE, Frölich M, Westendorp RG (2004). Thyroid status, disability and cognitive function, and survival in old age. JAMA.

[11] Grossman A, Weiss A, Koren-Morag N, Shimon I, Beloosesky Y, Meyerovitch J (2016). Subclinical thyroid disease and mortality in the elderly: a retrospective cohort study. Am J Med.

[12] Hyland KA, Arnold AM, Lee JS, Cappola AR (2013). Persistent subclinical hypothyroidism and cardiovascular risk in the elderly: the cardiovascular health study. J Clin Endocrinol Metab.

[13] Waring AC, Harrison S, Samuels MH, Ensrud KE, LeBlanc ES, Hoffman AR (2012). Osteoporotic fractures in men (MrOS) study. Thyroid function and mortality in older men: a prospective study. J Clin Endocrinol Metab.

[14] Pearce SHS, Razvi S, Yadegarfar ME, Martin-Ruiz C, Kingston A, Collerton J (2016). Serum thyroid function, mortality and disability in advanced old age: the Newcastle 85+ study. J Clin Endocrinol Metab.

[15] Suh S, Kim DK (2015). Subclinical Hypothyroidism and Cardiovascular Disease. Endocrinol Metab (Seoul).

[16] Sun J, Yao L, Fang Y, Yang R, Chen Y, Yang K, et al. Relationship between subclinical thyroid dysfunction and the risk of cardiovascular outcomes: a systematic review and Meta-analysis of prospective cohort studies. Int J Endocrinol. 2017. 10.1155/2017/8130796. 10.1155/2017/8130796 PMC5610794 29081800

[17] Iervasi G, Molinaro S, Landi P, Taddei MC, Galli E, Mariani F (2007). Association between increased mortality and mild thyroid dysfunction in cardiac patients. Arch Intern Med.

[18] Molinaro S, Iervasi G, Lorenzoni V, Coceani M, Landi P, Srebot V (2012). Persistence of mortality risk in patients with acute cardiac diseases and mild thyroid dysfunction. Am J Med Sci.

[19] Zhang M, Sara JD, Matsuzawa Y, Gharib H, Bell MR, Gulati R (2016). Clinical outcomes of patients with hypothyroidism undergoing percutaneous coronary intervention. Eur Heart J.

[20] Lee Y, Lim YH, Shin JH, Park J, Shin J (2018). Impact of subclinical hypothyroidism on clinical outcomes following percutaneous coronary intervention. Int J Cardiol.

[21] Razvi S, Shakoor A, VanderpumpM WJU, Pearce SHS (2008). The influence of age on the relationship between subclinical hypothyroidism and ischemic heart disease: a meta- analysis. J Clin EndocrinolMetab.

[22] Kim TH, Choi HS, Bae JC, Moon JH, Kim HK, Choi SH (2014). Subclinical hypothyroidism in addition to common risk scores for prediction of cardiovascular disease: a 10-year community-based cohort study. Eur J Endocrinol.

[23] Kovar FM, Fang IF, Perkmann T, Haslacher H, Slavka G, Födinger M (2015). Subclinical hypothyroidism and mortality in a large Austrian cohort: a possible impact on treatment?. Wien Klin Wochenschr.

[24] Andersen MN, Olsen AS, Madsen JC, Kristensen SL, Faber J, Torp-Pedersen C (2016). Long-term outcome in levothyroxine treated patients with subclinical hypothyroidism and concomitant heart disease. J Clin Endocrinol Metab.

[25] Shah R (2017). In older adults with subclinical hypothyroidism, levothyroxine did not improve symptoms or tiredness. Ann Intern Med.

[26] Grossman A, Feldhamer I, Meyerovitch J. Treatment with levothyroxin in subclinical hypothyroidism is associated with increased mortality in the elderly. Eur J Intern Med. 2018. 10.1016/j.ejim.2017.11.010. 10.1016/j.ejim.2017.11.010 29174213

[27] Jabbar A, Ingoe L, Pearce S, Zaman A, Razvi S. Thyroxine in acute myocardial infarction (ThyrAMI)-levothyroxine in subclinical hypothyroidismpost-acute myocardial infarction: study protocol for a randomised controlled trial. Trials. 2015. 10.1186/s13063-015-0621-5. 10.1186/s13063-015-0621-5 PMC4379597 25872532

